# Bone Scintigraphy in Staging of Newly Diagnosed Prostate Cancer in Regard of Different Risk Groups

**DOI:** 10.22038/AOJNMB.2019.35768.1242

**Published:** 2019

**Authors:** Sabina Sevcenco, Bernhard Grubmüller, Charlotte Sonneck-Koenne, Yasaman Ahmadi, Peter Knoll, Andreas Floth, Wolfgang Pokieser, Shahin Zandieh, Hans Christoph Klingler, Sharokh Shariat, Siroos Mirzaei

**Affiliations:** 1 Department of Urology, SMZ Ost, Donauspital, Vienna, Austria; 2 Department of Urology, University of Vienna, Austria; 3 Department of Nuclear Medicine with PET-Center, Wilhelminenspital, Vienna, Austria; 4 Department of Urology, Wilhelminenspital, Vienna, Austria; 5 Department of Pathology, Wilhelminenspital, Vienna, Austria; 6 Institute of Radiology and Nuclear Medicine, Hanusch Hospital, Vienna, Austria

**Keywords:** Bone scintigraphy, Gleason Score, Prostate cancer, Prostate specific antigen, PSA

## Abstract

**Objective(s):**

Prostate cancer (PC) is the most common cancer in men over 50 years of age. Bone scintigraphy is still performed in many institutions at the time of primary diagnosis. We aimed to evaluate the role of bone scan in the primary staging of PC in regard of different risk groups.

**Methods:**

A retrospective analysis of bone scans in 296 patients (mean age 64±6 y) acquired at the time of primary diagnosis was performed in our institution. The median prostate specific antigen (PSA) was 6.73 ng/ml, all patients had a Gleason score of >5.

**Results:**

Only 11/296 patients had a positive bone scan, 1 being in the intermediate risk group, 10 in the high-risk group and none in the low-risk group according to D’Amico classification.

**Conclusion:**

Our results support the few published studies that less than 10% of patients with newly diagnosed PC by biopsy would develop bone metastasis, all in the intermediate or high-risk groups. Therefore, a staging by bone scan can only be recommended in patients with intermediate or high-risk, or symptomatic patients only.

## Introduction

Prostate cancer is the most common cancer in men over 50 years of age, thus PSA screening is a widely-accepted method for early diagnosis. When verified by biopsy, routine screening includes, among others, bone scintigraphy to rule out bone metastases since they represent the main metastatic site in about 80% of prostate cancer with a significant contribution to the cost of care for those patients (1). 

The intense use of PSA testing has led to prostate cancer commonly being diagnosed at a very early stage (2). However, post-mortem examination reveals that ~70% of patients with metastatic prostate carcinomas have a high incidence of bone lesions (3). Existence of bone metastases is a very important prognostic factor (2) with high impact on decision making regarding intention to cure. A recent prospective study (4) of patients with newly diagnosed prostate cancer showed a prevalence of bone metastasis in only 13.7% (87 of 635 patients). No bone metastases were observed in patients with PSA value less than 10 ng/ml independently of clinical T-stage and Gleason score (n=212) and PSA value less than 20 ng/ml if T stage was less than T3 and Gleason sum less than 8 (n=97).

In another study the relationship between PSA levels and bone metastasis rate was observed and no bone metastases were seen in patients whose Gleason sums were less than five. In patients with Gleason sum score >5 and PSA level <15 ng/ml, there were also no bone metastases (5).

Similar results were recently reported in newly diagnosed PC patients with a PSA level of 10 ng/ml or lower and negative lymph nodes to have a very low risk of bone metastasis and therefore bone scans may not be necessary in these patient group (6). 

For the risk assessment there are different recommendations, taking into account PSA level, Gleason score based on microscopical pattern of prostate biopsy specimens and T-stage. In our institution we preferably use D’Amico risk stratification (7), with following classification, low-risk (stage T1c, T2a and PSA level ≤10 ng/mL and Gleason score ≤6), intermediate-risk (stage T2b or Gleason score of 7 or PSA level >10 and ≤20 ng/mL) and high-risk patients (stage T2c or PSA level >20 ng/mL or Gleason score ≥8). The aim of our study was to evaluate the role of bone scintigraphy in primary staging of PC in different risk groups.

## Methods

We obtained data of all the 517 patients who were diagnosed with PC from December 2004 to December 2012 from the department of urology of our hospital. The mean age was 64±6 years, median prostate specific antigen (PSA) was 6.73 ng/ml, all patients had a Gleason score of >5.

296/517 patients with newly diagnosed prostate cancer had initial bone scintigraphy in our department to exclude bone metastasis, irrespective of their PSA level or Gleason score. The remaining 221/517 patients had either bone scan in other institutions or had no bone scan of other clinical reasons, and therefore excluded from this retrospective study. Considering also the T stage only 50% of the patients with initial bone scan had local disease (T1 or T2). 

A whole body planar bone scintigraphy was performed 3-4 hours after intravenous injection of 740 MBq of Tc-99m diphosphonate on a Hawkeye Infinia (GE, Haifa, Israel) SPECT-CT gamma camera, 256×1024 matrix, with a LEHR collimator, 13 cm/min bed movement. Additional SPECT-(low dose) CT images were acquired with a T6-Symbia gamma camera (Siemens, Erlangen, Germany), in 29 cases with unclear focal increased tracer uptake in the spine (140 keV, 20 mAs).

The scintigraphic images were reported by two Nuclear Medicine physicians and the CT part of SPECT-CT was reported by one experienced radiologist, blinded to the detailed history of the patients. A focal intense uptake in planar images, without history of trauma, and in SPECT images a focal increased tracer uptake without benign degenerative changes in CT was considered as suspicious for bone metastasis. 

For this retrospective analysis, we applied the risk stratification as mentioned above (7). 

**Table 1:** Number of patients with positive bone scan in different risk groups

**Risk stratification according to D’Amico**	**Number of the patients**	**mean age (y)**	**Initial Bone scan positive (% of total patients)**
low-risk	30	63.8±4.3	0 (0%)
intermediate risk	30	64.6±5.9	1 (0.3%)
high-risk	236	64.1±6.3	10 (3.3%)
Total	296	64.0±6.3	11 (3.7%)

## Results

Table 1 indicates that 30 patients were classified in the low-risk group, 30 were in the intermediate risk group and 236 were in the high risk group according to D’Amico classification (7).

Eleven patients had a positive bone scan, one being in the intermediate risk group, ten in the high-risk group and none were in the low-risk group (Table1). The PSA level of these patients was in the range of 0, 15-10, 30 (mean value 5.75), with a Gleason score of 9 (1/11), 6 (1/11) and 7 in remaining cases. Out of 29 SPECT-CT scans only one patient (1/29) in the high-risk group demonstrated a suspicious lesion in the Lumbal spine, all other lesions in the planar whole body scans in this group were attributed to degenerative changes by SPECT-CT.

15% (43/296) of the patients had another bone scintigraphy 2.8 years after primary diagnosis, there of two with an initial positive result, because of increase of PSA level under therapy or because of newly skeletal pain. 84% (36/43) still had a negative bone scan, 16% (7/43) had a positive bone scan, while the patients with suspicious bone lesions were also initially in the high-risk group.

## Discussion

Bone scintigraphy has a high negative predictive value of up to 99.7% in patients with low serum PSA levels (8). However, the selection of patients for bone scan in the primary staging of prostate cancer is still an ongoing debating. 

On the other side, it is known that most patients with metastatic prostate cancer will develop bone metastasis and are best monitored by radionuclide bone scintigraphy (9). With the introduction of new techniques in molecular imaging like SPECT-CT the sensitivity and specificity of this method has improved significantly in the past years (10).

The recommendations for the use of bone scan in PC are mostly based on older studies without taking into account these new developments in molecular imaging and bone scan is not recommended if the PSA <20 ng/ml in asymptomatic patients (11). In our study SPECT-CT was used in selected cases with unclear enhanced tracer accumulation in skeleton. Additionally, some authors recommended the use of bone scan in symptomatic patients or if alkaline phosphatase levels are >90 U/l (12). In another recent study no correlation was found between pain intensity and both PSA value and presence of metastases and no correlation between localization of the symptoms and the site of bone metastases in bone scan. The authors described correlation between PSA value and both presence and number of metastases with a cutoff value of PSA of 10 ng/ml for negative bone scans (13).

In the present study 3.7% (11/296) of patients showed bone metastases at initial diagnosis, 1/11 being in intermediate and 10/11 in the high-risk group (Table 1). The follow-up bone scan remained negative in 84%, and showed positive results in 16% of patients, all of them being in the high-risk group. Therefore, one may conclude that only those patients initially in high-risk group, even with a negative bone scan, are at higher risk to develop bone metastasis, and should be followed up closely by bone scintigraphy. The patients at low risk have a good prognosis, and an initial bone scan in this group may not be useful, except in symptomatic patients. 

Additionally, we’ve to address new developments in targeted molecular imaging techniques with F18-Choline (14,15) and various PSMA (prostate-specific membrane antigen) Positron-Emission-Tomography (PET) scans (16,17) which will most probably be added to the routine clinical management of the patients with PC and recurrent disease in the near future with possible targeted therapeutic implications (17) and also have a significant effect on the use of bone scintigraphy in this patient group (18). 

The limitation of the present study is the low number of the patients. On the other side, to our best knowledge it is one of rare studies using risk groups for correlation with the bone lesions in bone scan, taking into account the PSA level, Gleason score and T-stage of the primary tumor (7, 19, 20). A correlation to risk group including different parameters seems to us to be a better surrogate than comparing only to one parameter, which may be hampered by other factors as i.e. unspecific elevated PSA level. Prospective multicentre trials using new imaging techniques (i.e. SPECT-CT) with higher sensitivity and specificity of bone scan in patients with prostate cancer are warranted to evaluate the role of these new techniques for staging of patients with prostate cancer, 

## Conclusion

Our results support the few published studies that less than 10% of patients with newly diagnosed PC by biopsy would develop bone metastasis, all in the intermediate or high-risk groups. Therefore, a staging by bone scan can only be recommended in patients with intermediate or high-risk, or symptomatic patients only. 
